# Acalabrutinib in Chinese patients with relapsed/refractory chronic lymphocytic leukemia: Primary analysis from an open-label, multicenter phase 1/2 trial

**DOI:** 10.1007/s00277-024-05978-4

**Published:** 2024-09-14

**Authors:** Shenmiao Yang, Haiwen Huang, Keshu Zhou, Xielan Zhao, Yanqiu Han, Lindong Li, Yujie Wang, Xiaofeng Liu, Jianyong Li

**Affiliations:** 1 Peking University Peoples Hospital, Peking University Institute of Hematology, Beijing, China; 2 The First Affiliated Hospital of Soochow University, Suzhou, China; 3 Affiliated Cancer Hospital of Zhengzhou University, Henan Cancer Hospital, Zhengzhou, China; 4 Xiangya Hospital Central South University, Changsha, China; 5 The Affiliated Hospital of Inner Mongolia Medical University, Hohhot, China; 6 AstraZeneca, Shanghai, China; 7 Department of Hematology, The First Affiliated Hospital of Nanjing Medical University, Nanjing, China; 8 Collaborative Innovation Center for Cancer Personalized Medicine, Jiangsu Province Hospital, Nanjing, People’s Republic of China

**Keywords:** Chronic Lymphocytic Leukemia, Acalabrutinib, Refractory/Relapsed

## Abstract

Acalabrutinib is a highly selective Bruton tyrosine kinase inhibitor approved in the United States and Europe for chronic lymphocytic leukemia (CLL) based on phase 3 trials with limited representation of Asian populations. This phase 1/2 trial evaluates acalabrutinib in Chinese adults with relapsed/refractory (R/R) CLL receiving acalabrutinib 100 mg twice daily in 28-day cycles until disease progression or treatment discontinuation due to adverse events (AEs) presenting substantial clinical risk. The primary endpoint was blinded independent central review (BICR)-assessed overall response rate (ORR). A total of 60 patients from 20 sites in China received acalabrutinib (median age 62 years; median 1 prior therapy line; 21.7% with del(17p) and/or *TP53* mutation; 51.7% with unmutated IGHV). Median total treatment duration was 19.4 months (range 0.6–28.2) with 53 patients (88.3%) receiving acalabrutinib at data cutoff; median study follow-up was 20.2 months. BICR-assessed ORR was 85.0% (95% CI, 73.4–92.9). Median duration of response, progression-free survival (PFS), and overall survival (OS) were not reached. Estimated 12-month and 18-month PFS rates were 91.5% (95% CI, 80.9–96.4) and 78.8% (95% CI, 60.9–89.2); OS rates were both 96.7% (95% CI, 87.3–99.2). AEs of grade ≥ 3 occurred in 25 patients (41.7%), most commonly decreased neutrophil count (13.3%, n = 8), pneumonia (6.7%, n = 4), and upper respiratory tract infection (6.7%, n = 4). AEs led to treatment discontinuation in 2 patients (paraneoplastic pemphigus; rectal neoplasm). This study demonstrated high ORR in acalabrutinib-treated Chinese patients with R/R CLL with no unexpected safety concerns. This trial is registered on ClinicalTrials.gov (NCT03932331).

**Supplementary Information:**

The online version contains supplementary material available at 10.1007/s00277-024-05978-4.

## Introduction

Chronic lymphocytic leukemia (CLL) is the most common type of leukemia in Western countries, with an incidence of approximately 4.2/100,000 per year [1]. The incidence in China is much lower, estimated at 0.3/100,000 [2]. However, global trends from 1990 to 2017 have demonstrated that the greatest increase in CLL incidence occurred in East Asia, suggesting a growing burden in this region [3].

Treatment of CLL has been shifting from chemoimmunotherapy (CIT) only to the use of small molecule inhibitors, such as Bruton tyrosine kinase (BTK) inhibitors and B-cell lymphoma 2 (BCL2) inhibitors [4]. Acalabrutinib is a highly selective BTK inhibitor with minimal off-target activity approved in the US and Europe for the treatment of CLL [5, 6]. The benefit of acalabrutinib versus CIT was demonstrated by a significant improvement in progression-free survival (PFS) among patients with treatment-naive (TN) CLL (ELEVATE-TN) and R/R CLL (ASCEND) [7–9]. However, these pivotal studies supporting the approval of acalabrutinib were conducted predominantly in Europe and North America, with limited representation from Asian populations [7, 8].

This phase 1/2 trial, along with the ASCEND trial, led to the approval of acalabrutinib in China for the treatment of patients with R/R CLL and small lymphocytic lymphoma (SLL). We report the results of the single-agent phase 1/2 trial, which is the first to assess the efficacy and safety of acalabrutinib exclusively in Chinese patients with R/R CLL.

## Methods

### Study design and patients

This phase 1/2 open-label study was conducted at 20 sites in China and divided into 2 parts. The phase 1 portion, reported previously, assessed the safety, tolerability, and pharmacokinetics of acalabrutinib in Chinese patients with R/R B-cell malignancies and included 4 patients with CLL (NCT03932331) [10]. The phase 2 portion, reported herein, evaluated the clinical efficacy, safety, and tolerability in subjects with R/R CLL. Patients aged ≥ 18 years at the time of study entry were eligible for inclusion if they had active CLL as per the International Workshop on Chronic Lymphocytic Leukemia (iwCLL) 2018 criteria that required treatment [11], had received 1 or more prior systemic therapies for CLL, and had an Eastern Cooperative Oncology Group (ECOG) performance status of ≤ 2. Key exclusion criteria included significant cardiovascular disease (eg, uncontrolled or symptomatic arrhythmias, congestive heart failure, or myocardial infarction within 6 months of screening) and known central nervous system involvement of lymphoma/leukemia or leptomeningeal disease. Full inclusion and exclusion criteria are provided in Online Resource 1.

Patients received acalabrutinib 100 mg orally twice daily in 28-day cycles until disease progression, unacceptable toxicity, or any other discontinuation criteria were met. A dose delay of up to 28 days was permitted in the event of potentially drug-related toxicity of grade ≥ 3 in severity. Study treatment was discontinued in the event of toxicity requiring the postponement of dosing lasting > 28 days unless reviewed and approved by the sponsor. In the event of toxicity, dose modification was allowed as described in Online Resource 2. Radiologic tumor assessment was performed at baseline and every 12 weeks (± 7 days), with on-treatment radiologic assessments occurring on day 1 of cycle 4, day 1 of cycle 7, then every 3 cycles until cycle 25, and every 24 weeks thereafter. A safety follow-up was performed 30 days (+ 7 days) following the last dose of therapy. Patients with disease progression were followed-up every 12 weeks to assess survival and the use of alternative anticancer therapy until death or loss to follow-up. Patients who discontinued therapy for reasons other than disease progression remained in the study for post-treatment follow-up.

All patients provided written informed consent. An institutional review board and independent ethics committee reviewed and approved the protocol before study initiation. The study was conducted according to the principles from international guidelines including the Declaration of Helsinki, Council for International Organizations of Medical Sciences International Ethical Guidelines, and applicable International Conference on Harmonization Good Clinical Practice guidelines.

### Endpoints

The primary endpoint was overall response rate (ORR) as assessed by blinded independent central review (BICR) per the iwCLL 2018 criteria [11]. Secondary endpoints included investigator-assessed ORR and time to response (TTR) and BICR- and investigator-assessed duration of response (DOR), progression-free survival (PFS), and time to next treatment (TTNT). ORR was defined as the proportion of patients who achieved a nodular partial response (nPR), partial response (PR), complete response (CR), or CR with incomplete marrow recovery (CRi) as best overall response. Although the study used iwCLL 2018 response assessment criteria for CLL, ORR including patients with a best overall response of partial response with lymphocytosis (PRL) was also assessed and denoted as ORR + PRL. PRL was defined as the presence of lymphocytosis plus ≥ 50% reduction in lymphadenopathy and/or in spleen or liver enlargement, plus having met one of the PR criteria for platelets or hemoglobin. DOR was defined as the interval from the first documentation of objective response to the earlier of the first documentation of objective disease progression by the BICR or death from any cause. PFS was defined as the interval from start of acalabrutinib therapy to the earlier of the first documentation of objective disease progression by the BICR or death from any cause. TTR was defined as the interval between the date of first dose and date of initial documentation of a response. TTNT was defined as the interval from the start of acalabrutinib therapy to the initiation of non-protocol–specific treatment for CLL or death due to any cause, whichever was earlier. OS was defined as the interval from the start of acalabrutinib therapy to death from any cause. Subgroup analyses were performed based on genetic abnormalities (ie, del(17p), del(11q), IGHV mutational status, and *TP53* mutational status).

Safety endpoints included a standard collection of adverse event (AE) data and other study variables. Safety assessments included vital signs, 12-lead electrocardiogram (ECG), urinalysis, and blood sampling for hematology, clinical chemistry, and coagulation parameters. Treatment-emergent AEs (TEAEs); drug-related TEAEs; serious TEAEs (also referred to as SAEs); TEAEs leading to treatment discontinuation, dose delay, or dose modification; TEAEs of special interest (AESIs); and events of clinical interest (ECIs) were recorded. TEAE was defined as an AE that occurred either before the first administration of study drug but worsened at or after the first dose of study treatment or that occurred at or after the first dose of study treatment, throughout the treatment period, and within 30 days after the last dose of study treatment. All AEs were recorded until 30 days after the last dose of acalabrutinib or the start of a new anticancer therapy, whichever occurred first. AEs were graded according to Common Terminology Criteria for Adverse Events (CTCAE) version 5.0. AESIs included ventricular arrhythmias (eg, ventricular tachycardia, ventricular arrhythmia, and ventricular fibrillation) as well as ventricular extrasystoles. ECIs included cardiac events, cytopenias, hemorrhage, and hypertension. AEs were assessed by the investigator as to whether they were related to study treatment.

### Statistical analysis

Sample size was calculated to achieve 90% power to detect the difference between a null hypothesis ORR of 70% and an alternative ORR of 88% based on an exact binomial test with a nominal 1-sided significance level of 2.5%. The safety analysis set included all patients who received at least 1 dose of acalabrutinib. The tumor response analysis set included patients who received at least 1 dose of acalabrutinib and had a relevant baseline tumor assessment.

Primary analysis of ORR was conducted on the tumor response analysis set with the ORR and corresponding 95% confidence interval (CI) based on the Clopper–Pearson exact method. Analyses of DOR, PFS, and OS were estimated using the Kaplan–Meier (K–M) method. Best overall response, TTR, and TTNT were summarized using descriptive statistics.

## Results

### Patients

A total of 84 patients were screened for inclusion in the R/R CLL cohort in phase 2 of the study, of whom 60 patients were enrolled at 20 sites in China from August 17, 2020 to December 24, 2021 and received acalabrutinib treatment. Patients were predominantly male and had a median age of 62 years (range, 33–77) (Table 1). At the time of data cutoff (December 22, 2022), 53 (88.3%) patients remained on acalabrutinib therapy and 7 (11.7%) had discontinued study treatment. The most common reasons for treatment discontinuation were progressive disease (n = 3; 5.0%) and AEs (n = 2; 3.3%). The median time on study was 20.2 months (range 1.1–28.2). Protocol deviations due to COVID-19 included 3 (5.0%) patients with temporary withholding of study drug for ≥ 7 days, and 2 (3.3%) patients who did not have tumor assessments at cycle 4 and cycle 7.

**Table 1:** Demographics and baseline characteristics

	Acalabrutinib (*N* = 60)
Age, median (range), y	62 (33–77)
Female	19 (31.7)
ECOG PS ≤ 1	58 (96.7)
Bulky lymph nodes	
≥ 5 cm	22 (36.7)
≥ 10 cm	3 (5.0)
Rai stage 3 or 4	29 (48.3)
Prior medical history	
Hypertension	17 (28.3)
Cardiac disorders	17 (28.3)
Atrial fibrillation	2 (3.3)
Prior lines of therapy, median (range)	1 (1 − 4)
High-risk features	
IGHV unmutated	31 (51.7)
del(17p)	7 (11.7)
* TP53* mutation	7 (11.7)
del(17p) and/or *TP53* mutation	13 (21.7)

Values are n (%) unless otherwise noted

*ECOG PS* Eastern Cooperative Oncology Group performance status, *IGHV* immunoglobulin heavy chain variable region genes

### Efficacy

**Fig. 1:**
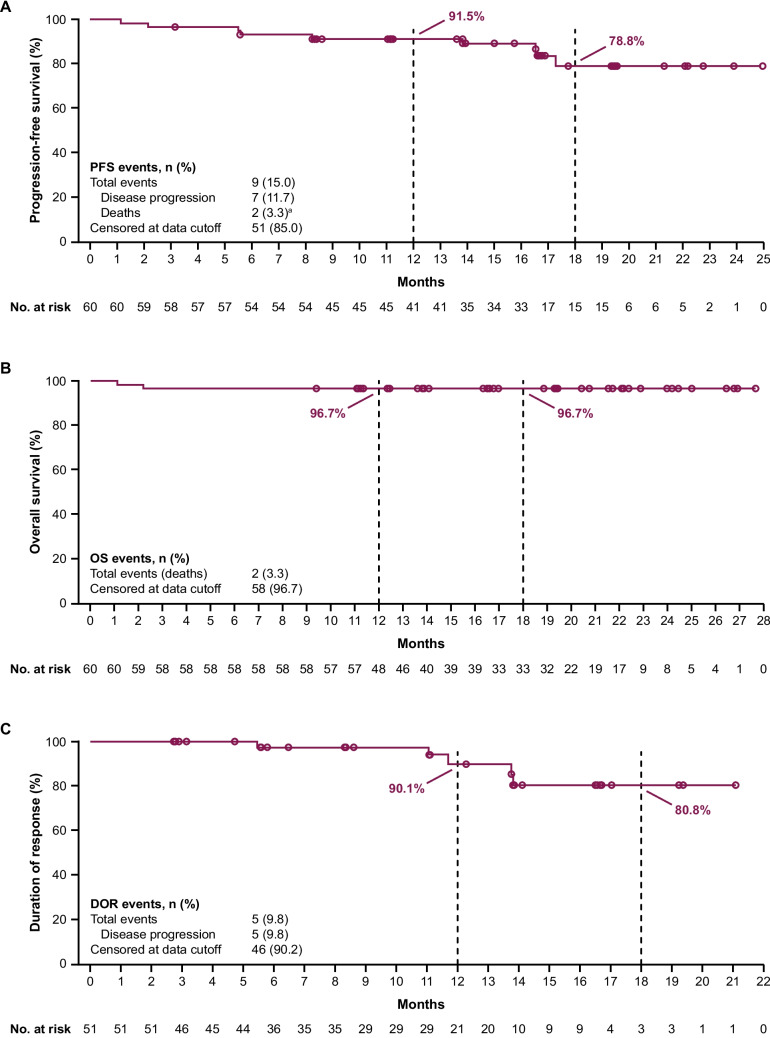
Progression-free survival as assessed by BICR (**A**), overall survival (**B**), and duration of response as assessed by BICR (**C**). ^a^Two deaths were reported during the study: 1) cardiac arrest in a 73-year-old patient on study day 34 (2 days after the last dose of study drug) and possibly related to study drug, and 2) hemorrhagic shock in a 55-year-old patient on study day 66 (50 days after discontinuing study drug) and unclear about the relationship to study treatment. BICR, blinded independent central review; DOR, duration of response; OS, overall survival; PFS, progression-free survival

The estimated 12- and 18-month PFS rates as assessed by BICR were 91.5% (95% CI, 80.9–96.4) and 78.8% (95% CI, 60.9–89.2), respectively (Fig. 1A). PFS rates were higher when assessed by investigators (12-month: 95.0% [95% CI, 85.2–98.3]; 18-month: 87.2% [95% CI, 69.1–95.0]) (Online Resource 3A). The BICR-assessed PFS rates at 12 months were generally consistent across all pre-specified subgroups (Online Resource 4). A total of 3 (5.0%) patients had TTNT-related events, and the K–M estimates of the proportion of patients who did not start the next anticancer treatment at 12 months and 18 months were both 95.0% (95% CI, 85.3–98.4), with median TTNT not reached. The median OS was also not reached; the 12- and 18-month OS rates were both 96.7% (95% CI, 87.3–99.2) (Fig. 1B).

**Fig. 2:**
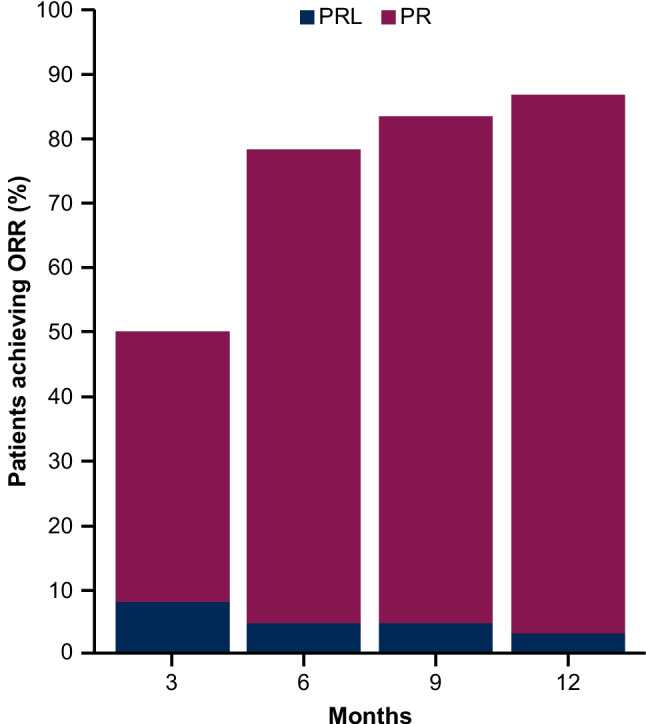
Cumulative percentage of patients achieving ORR during first 12 months as assessed by BICR. BICR, blinded independent central review; PR, partial response; PRL, partial response with lymphocytosis

**Fig. 3:**
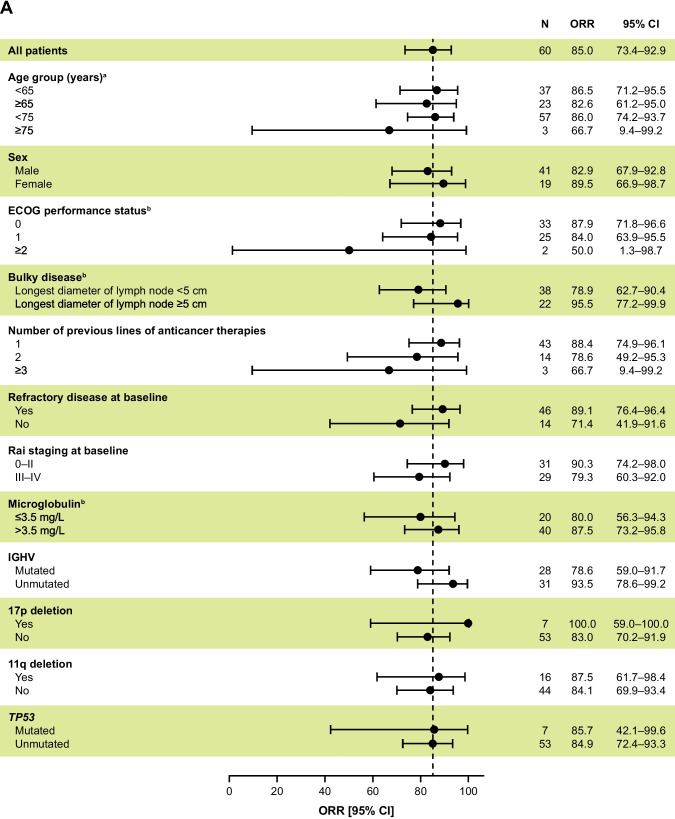

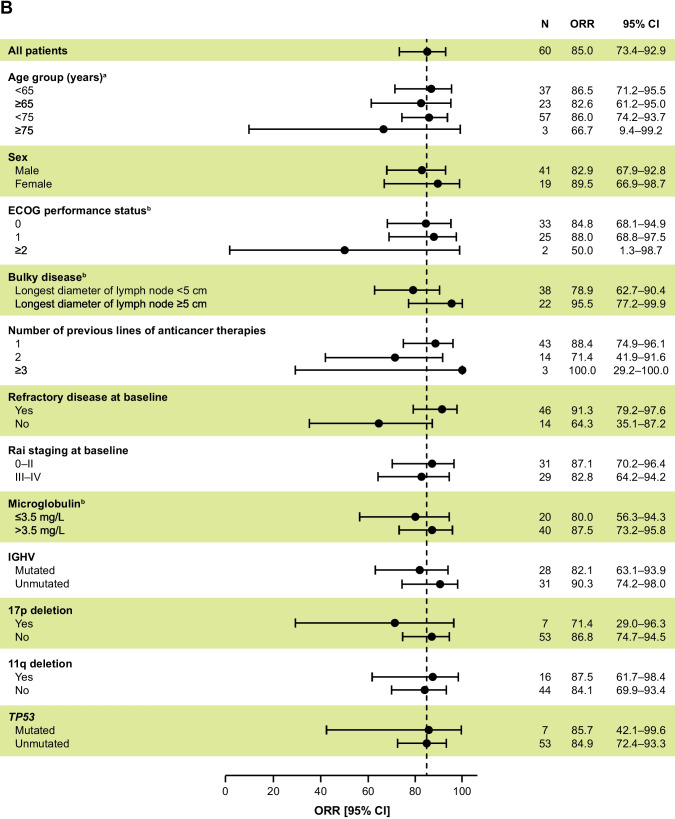
Subgroup analysis of ORR by BICR (**A**) and investigator (**B**) assessment per iwCLL criteria (tumor response set). BICR, blinded independent central review; CI, confidence interval; ECOG, Eastern Cooperative Oncology Group; IGHV, immunoglobulin heavy chain variable region genes; iwCLL, International Workshop on Chronic Lymphocytic Leukemia; ORR, overall response rate. ^a^Age when informed consent was signed; ^b^At baseline

According to BICR and investigator assessments, the ORR (CR + PR) was 85.0% (95% CI, 73.4–92.9) (Table 2). BICR and investigator assessments identified CR in 0 and 1 (1.7%) patient and PR in 51 (85.0%) and 50 (83.3%) patients, respectively. Additionally, PRL was identified in 1 (1.7%) and 3 (5.0%) patients in the BICR and investigator assessments, respectively. ORR + PRL was 86.7% and 90.0% as assessed by BICR and investigators, respectively. The cumulative percentage of patients reaching PR and PRL as assessed by BICR during the first 12 months of treatment is shown in Fig. 2, with 86.7% achieving PR + PRL. The BICR- and investigator-assessed 12-month DOR rates were 90.1% (95% CI, 71.3–96.8) and 100% (95% CI, 100.0–100.0), respectively (Fig. 1C and Online Resource 3B). Median DOR was not reached with either BICR or investigator assessment. The ORR across the prespecified subgroups was generally consistent with the primary efficacy analysis as assessed by BICR (Fig. 3A) and investigators (Fig. 3B), including those with high-risk genomic features (ie, IGHV mutation status, 17p deletion, 11q deletion, and *TP53* mutation). The median TTR as assessed by BICR was 4.63 months (range, 2.7–16.6), which was comparable to the TTR as assessed by investigators (median, 5.52 months; range, 2.7–22.2).

**Table 2:** Efficacy results by BICR and investigator assessment

	Acalabrutinib (*N* = 60)	
	BICR	Investigator
ORR^a^, n (%) [95% CI]	51 (85.0) [73.4–92.9]	51 (85.0) [73.4–92.9]
ORR + PRL^b^, n (%) [95% CI]	52 (86.7) [75.4–94.1]	54 (90.0) [79.5–96.2]
Best overall response, n (%)^c^		
CR	0	1 (1.7)
PR	51 (85.0)	50 (83.3)
PRL	1 (1.7)	3 (5.0)
SD	6 (10.0)	4 (6.7)
PD	0	0
Median DOR^d^, months (95% CI)	NR (NE–NE)	NR (NE–NE)
Estimated 12-month DOR rate, % (95% CI)	90.1 (71.3–96.8)	100 (100–100)
Estimated 18-month DOR rate, % (95% CI)	80.8 (58.8–91.8)	92.9 (59.1–99.0)

*BICR* blinded independent central review, *CI* confidence interval, *CR* complete response, *DOR* duration of response, *iwCLL* International Workshop on Chronic Lymphocytic Leukemia, *NR* not reached, *NE* not evaluable, *ORR *overall response rate, *PD* progressive disease, *PR* partial response, *PRL* partial response with lymphocytosis, *SD *stable disease

^a^ORR (CR + PR) presented with the corresponding 95% CI based on Clopper–Pearson exact method

^b^Although response categories were based on iwCLL 2018 criteria, the analysis also included the category of PRL as described in Methods

^c^Two patients were not evaluable for response

^d^DOR by BICR and by investigator were estimated using the Kaplan–Meier method

### Safety

In the safety analysis set, the median total treatment duration was 19.4 months (range, 0.6–28.2), with a median relative dose intensity of 99.9% (defined as the percentage of the actual dose delivered relative to the intended dose through treatment discontinuation). TEAEs of any grade overall were reported in 58 patients (96.7%), with 25 patients (41.7%) reporting an AE of grade 3 or higher (Table 3). The most frequent TEAEs of any grade or grade ≥ 3 are shown in Table 4. The most common infections reported were upper respiratory tract infection (n = 11, any grade) and pneumonia (n = 9, any grade). Four (6.7%) patients had COVID-19, 1 patient had COVID-19 pneumonia, and 1 patient was suspected of COVID-19, though none of these events were grade 3 or higher and no COVID-19 deaths were reported. Other TEAEs of interest for BTK inhibitors were headache (n = 10 [16.7%]), diarrhea (n = 9 [15.0%]), myalgia (n = 2 [3.3%]), and arthralgia (n = 1 [1.7%]). Treatment discontinuation due to AEs was reported in 2 patients (1 with paraneoplastic pemphigus and 1 with rectal neoplasm). Dose interruptions due to AEs occurred in 13 patients, most commonly due to COVID-19 (5.0%), neutrophil count decreased (3.3%), and platelet count decreased (3.3%). Nine patients (15.0%) reported SAEs (Table 3 and Online Resource 5).

**Table 3:** Treatment-emergent adverse events in any category (safety analysis set)

TEAE category	No. (%) of patients (*N* = 60)^a^
Any TEAE	58 (96.7)
Any TEAE grade ≥ 3	25 (41.7)
Any TEAE causally related to treatment^b^	53 (88.3)
Any TEAE causally related to treatment grade ≥ 3^b^	23 (38.3)
Any serious TEAE	9 (15.0)
Any serious TEAE causally related to treatment^b^	8 (13.3)
Any TEAE with outcome of death	1 (1.7)
Any TEAE causally related to treatment with outcome of death^b^	1 (1.7)
Any TEAE leading to dose interruption	13 (21.7)
Any TEAE leading to dose modification	1 (1.7)
Any TEAE leading to discontinuation of treatment	2 (3.3)

*TEAE* treatment-emergent adverse event

^a^Patients with multiple events in the same category were counted only once in that category. Patients with events in more than one category were counted once in each of those categories

^b^Causally related to treatment, as assessed by the investigator. Missing assessments of causality were counted as related

**Table 4:** Treatment-emergent adverse events of any grade (in ≥ 10% of patients) or grade ≥ 3 (in ≥ 5% of patients)

TEAEs, n (%)	Acalabrutinib (*N* = 60)	
	Any Grade	Grade ≥ 3
Neutrophil count decreased	24 (40.0)	8 (13.3)
Platelet count decreased	20 (33.3)	3 (5.0)
Hemoglobin decreased	14 (23.3)	3 (5.0)
Anemia	13 (21.7)	3 (5.0)
Upper respiratory tract infection	11 (18.3)	4 (6.7)
Headache	10 (16.7)	0
Diarrhea	9 (15.0)	1 (1.7)
Hematocrit decreased	9 (15.0)	3 (5.0)
Pneumonia	9 (15.0)	4 (6.7)
Petechiae	8 (13.3)	0
Rash	8 (13.3)	0
Red blood cell count decreased	8 (13.3)	2 (3.3)
Alanine aminotransferase increased	6 (10.0)	0
Aspartate aminotransferase increased	6 (10.0)	0
Hyperuricemia	6 (10.0)	0
Lymphocyte count increased	3 (5.0)	3 (5.0)

*TEAE* treatment-emergent adverse event

Two fatal AEs were reported during the course of the study. One death was determined to be due to cardiac arrest in a 73-year-old male that occurred on day 34 (2 days after the last dose of study drug). The patient’s relevant cardiorespiratory medical history included sinusitis, bronchiectasis, bronchitis, abnormal electrocardiogram (ECG) T-wave, high-density lipoprotein cholesterol decreased, hyperglycemia, hyperlipidemia, low-density lipoprotein cholesterol decreased, and abnormal ECG QRS complex. The investigator reported a reasonable possibility of a causal relationship with study drug. The second death involved hemorrhagic shock in a 55-year-old male with a history of hypertension. In this patient, acalabrutinib was discontinued on day 16 of study treatment due to development of grade 4 paraneoplastic pemphigus, which was assessed by the investigator to be possibly related to study treatment; the patient died on day 66 (50 days after discontinuing acalabrutinib) with no definitive association between the cause of death (hemorrhagic shock) and paraneoplastic pemphigus established.

Ventricular arrhythmia occurred in 2 (3.3%) patients, and asymptomatic ventricular extrasystoles occurred in 3 (5.0%) patients. One of these patients had both ventricular arrhythmia and asymptomatic ventricular extrasystoles. All these events were determined to be asymptomatic grade 1 or 2 events and did not result in treatment interruption, reduction, or discontinuation. Among these 4 patients with one or both events, 3 had a prior history of cardiovascular disorders (including hypertension [n = 2], extrasystoles [n = 2], aortic valve incompetence, atherosclerosis, and diastolic dysfunction [n = 1 each]). Among ECIs, infection occurred in 36 (60.0%) patients, neutropenia in 24 (40.0%) patients, hypertension in 3 (5.0%) patients, and hemorrhage in 24 (40.0%) patients. Among the 3 patients with hypertension, 1 patient had a prior history of cardiovascular disorders (cardiomegaly, left ventricular hypertrophy, atherosclerosis, and prolonged PR interval and ST segment abnormalities detected on electrocardiogram). All hemorrhage events were grade 1 or 2 with the most common events (occurring in > 2 patients) being petechiae (n = 8), ecchymosis (n = 4), hemorrhage (n = 3), subcutaneous hemorrhage (n = 3), and skin hemorrhage (n = 3). There were no cases of atrial fibrillation/flutter, major hemorrhage, second primary malignancies, or tumor lysis syndrome reported within 30 days of the last dose of study drug.

## Discussion

This is the first clinical trial evaluating the efficacy and safety of acalabrutinib in a Chinese patient population with R/R CLL. In this population, acalabrutinib treatment achieved a high ORR of 85% when assessed by either BICR or investigators. Furthermore, median DOR, PFS, and OS were not reached during the course of the study, demonstrating durability of these efficacy results. Continuous treatment with acalabrutinib was well tolerated, toxicities were manageable, and no unexpected safety observations were detected in this Chinese patient population.

This study aimed to determine whether the safety and efficacy of acalabrutinib in a Chinese patient population was comparable to previously reported global phase 3 trial results that included predominantly Caucasian patients with R/R CLL [8, 12]. This is particularly important, as evidence has demonstrated notable differences in the frequency of common genetic mutations and chromosomal abnormalities between Asian and European patients with CLL [13, 14]. For example, notably higher rates of del(11q), *MYD88*, and *TP53* have been observed among Asian patients with CLL compared with those of European descent [13, 14]. Establishing the safety of acalabrutinib in the Chinese patient population will also be important given differences in the prevalence of certain cardiac disorders and risk factors when compared with European populations. Data from the Global Burden of Disease Study demonstrated a higher prevalence rate (per 100,000) of hypertensive heart disease in East Asia (including China) versus Western Europe (189.0 vs 108.9, respectively), though there was a lower prevalence rate of atrial fibrillation and flutter in East Asia (551.6) versus Western Europe (749.7) [15]. Among cardiovascular risk factors, the prevalence rate for diabetes was 6157.7 in China compared with 5378.6 in Western Europe [16]. The efficacy of acalabrutinib in an Asian patient population with R/R CLL/SLL was previously demonstrated in a phase 1 Japanese trial [17]; although ORR reached 100% in the trial (CR + PR + PRL), the sample size was small (n = 9), highlighting the need for additional, more robust studies.

While comparing results from separate clinical trials can be problematic, efficacy outcomes in this study were, nonetheless, consistent with results observed in previous global trials that evaluated acalabrutinib for R/R CLL [8, 12]. In the ASCEND trial (with 4.5% of participants from the Asia region), at a median follow-up of 16.1 months, independent review committee–assessed ORR was 81% with acalabrutinib monotherapy [8] compared with 85% in this study. Median PFS and OS were not reached in ASCEND, and the OS rate at 12 months was 94% (compared with 96.7% in this study). A separate phase 1/2 study (with no participating sites in Asia) evaluating acalabrutinib monotherapy in patients with R/R CLL/SLL reported by Byrd and colleagues similarly demonstrated an ORR of 94% while median DOR and PFS were not reached with a median 41 months of treatment [12, 18]. The nature and frequency of AEs reported in this study were also comparable to findings from previous acalabrutinib clinical trials in patients with R/R CLL [8, 12].

Although the present study spanned the COVID-19 pandemic, no substantial impacts on safety or efficacy due to COVID-19 were noted. There were 4 patients with COVID-19 infection, 1 patient with COVID-19 pneumonia, and 1 patient suspected of having COVID-19. However, none of these events was assessed as grade 3 or higher and no deaths due to COVID-19 were reported.

Acalabrutinib represents 1 of 4 covalent BTK inhibitors approved in China for CLL (ibrutinib, zanubrutinib, and orelabrutinib). An overview of the efficacy of these 4 BTK inhibitors from clinical trials (phase 1/2/3) of Chinese patients with R/R CLL/SLL is shown in Online Resource 6 [19–21]. From these trials, ORR (CR + PR + PRL) was comparable among the second-generation BTK inhibitors (acalabrutinib [87%], zanubrutinib [85%], and orelabrutinib [93%]) while the ORR for ibrutinib (68%) was numerically lower, with median follow-up ranging from 15 to 32 months. Prior studies have demonstrated improved responses with longer duration of exposure to BTK inhibitors [21, 22]. Each BTK inhibitor demonstrated high OS, PFS, and event-free survival rates, suggesting an important role of BTK inhibitors for the treatment of R/R CLL among the Chinese patient population. Among cardiovascular AEs of clinical interest to BTK inhibitors, rates of hypertension were 5.0% for acalabrutinib and orelabrutinib, 5.8% for ibrutinib, and 9.9% for zanubrutinib. Atrial fibrillation was reported only with ibrutinib (5.8%); no cases were identified with the other BTK inhibitors.

The recent availability of head-to-head trial results can help differentiate the safety and efficacy among BTK inhibitors. The phase 3 ELEVATE-RR study compared acalabrutinib versus ibrutinib in patients with R/R CLL and demonstrated comparable PFS but significantly lower frequency of atrial fibrillation (any grade) and hypertension (any grade and grade ≥ 3) compared with ibrutinib [23]. The ALPINE study compared zanubrutinib versus ibrutinib for R/R CLL/SLL and demonstrated significantly lower risk of disease progression or death with zanubrutinib along with a lower incidence of atrial fibrillation, although the incidence of hypertension (any grade and grade ≥ 3) was similar between the treatment groups [24].

This study has some limitations worth mentioning. The small cohort size in this phase 1/2 study limits the reliability of subgroup analyses. As PFS and OS are the more important endpoints for clinical trials of patients with CLL, longer follow-up is needed to fully assess the efficacy of acalabrutinib in this patient population with R/R CLL. Finally, the study was conducted during the COVID-19 pandemic and resulted in some protocol deviations, such as temporary withholding of study drug and missed tumor assessments. However, these deviations were unlikely to have an impact on patient safety and outcomes and all 60 patients were included in the safety analysis and tumor response analysis sets.

## Conclusion

This is the first study to evaluate the efficacy and safety of acalabrutinib for the treatment of R/R CLL in a Chinese patient population. With a median time on study of 20.2 months, acalabrutinib demonstrated a high ORR with durable responses. Efficacy results were similar to trials of R/R CLL in Caucasian populations [8].

## Supplementary Information

Below is the link to the electronic supplementary material.

Supplementary file1 (DOCX 303 KB)

## Data Availability

Data underlying the findings described in this manuscript may be obtained in accordance with AstraZeneca’s data sharing policy described at https://astrazenecagrouptrials.pharmacm.com/ST/Submission/Disclosure. Data for studies directly listed on Vivli can be requested through Vivli at www.vivli.org. Data for studies not listed on Vivli can be requested through Vivli at https://vivli.org/members/enquiries-about-studies-not-listed-on-the-vivli-platform/. AstraZeneca Vivli member page is also available outlining further details: https://vivli.org/ourmember/astrazeneca/.
