# Supplementary material for: Acalabrutinib in Chinese patients with relapsed/refractory chronic lymphocytic leukemia: Primary analysis from an open-label, multicenter phase 1/2 trial
Source: Ann Hematol. 2024 Sep 14;104(1):701–12. doi: 10.1007/s00277-024-05978-4 (PMC11868377; doi:10.1007/s00277-024-05978-4)
Supplement: Supplementary file 1 — Supplementary file1 (DOCX 303 KB) [file 277_2024_5978_MOESM1_ESM.docx]

**Acalabrutinib in Chinese Patients With Relapsed/Refractory Chronic Lymphocytic Leukemia: Primary Analysis From an Open-Label, Multicenter Phase 1/2 Trial**

***Annals of Hematology***

Shenmiao Yang, MD^1^; Haiwen Huang, MD^2^; Keshu Zhou, MD^3^; Xielan Zhao, MD^4^; Yanqiu Han, MD^5^; Lindong Li, MD^6^; Yujie Wang, MD^6^; Xiaofeng Liu, MS^6^; Jianyong Li, MD^7^

^1^Peking University Peoples Hospital, Peking University Institute of Hematology, Beijing, China; ^2^The First Affiliated Hospital of Soochow University, Suzhou, China; ^3^Affiliated Cancer Hospital of Zhengzhou University, Henan Cancer Hospital, Zhengzhou, China; ^4^Xiangya Hospital Central South University, Changsha, China; ^5^The Affiliated Hospital of Inner Mongolia Medical University, Hohhot, China; ^6^AstraZeneca, Shanghai, China; ^7^The First Affiliated Hospital of Nanjing Medical University, Nanjing, China

**Corresponding Author:**

Jianyong Li, MD

Director of Lymphoma Center, Department of Hematology

The First Affiliated Hospital of Nanjing Medical University,

Jiangsu Province Hospital, Collaborative Innovation Center for Cancer Personalized Medicine

Nanjing, People’s Republic of China

Phone: +86 13951877733

E-mail: lijianyonglm@126.com

**Online Resource 1.** Inclusion and Exclusion Criteria

| **Inclusion criteria for chronic lymphocytic leukemia (CLL) cohort in phase 2** |
| --- |
| 1. Capable of providing signed informed consent, which includes compliance with the requirements and restrictions listed in the informed consent form and study protocol 2. Provision of signed and dated, written informed consent form prior to any mandatory study-specific procedures, sampling, and analyses 3. Chinese patients at least 18 years of age at the time of study entry 4. Eastern Cooperative Oncology Group (ECOG) performance status ≤2 5. Life expectancy of ≥3 months 6. Adequate hematologic function defined as absolute neutrophil count (ANC) ≥0.75 × 10^9^/L and platelet count ≥50 ×10^9^/L and, for patients with disease involvement in bone marrow, ANC ≥0.50 × 10^9^/L and platelet count ≥30 × 10^9^/L. Patients must be without growth factor support and platelet transfusion support 7 days before assessment. 7. Adequate organ function defined as aspartate aminotransferase and alanine aminotransferase ≤2 × upper limit of normal (ULN); total bilirubin ≤1.5 × ULN except in patients with documented Gilbert’s disease, for whom total bilirubin must be ≤2.5 × ULN; and estimated creatinine clearance of ≥50 mL/min, calculated using the Cockcroft and Gault formula: [(140 − age) • mass (kg) / (72 • creatinine mg/dL) • 0.85 if female] 8. Presence of radiographically measurable lymphadenopathy or extranodal lymphoid malignancy (for CLL: ≥1 nodal lesion >2.0 cm in the longest diameter as assessed by computed tomography scan) 9. Diagnosis of CLL that meets International Workshop on Chronic Lymphocytic Leukemia (iwCLL) diagnostic criteria [1]:    1. Monoclonal B cells (either kappa or lambda light chain restricted) that are clonally co-expressing ≥1 B-cell marker (CD19, CD20, or CD23) and CD5    2. Prolymphocytes may comprise <55% of blood lymphocytes    3. Presence of ≥5 × 10^9^ B lymphocytes/L (5000/μL) in peripheral blood (at any point since the initial diagnosis) 10. Must have received ≥1 prior systemic therapies for CLL. Note: Single-agent steroids or localized radiation are not considered a prior line of therapy. If a single-agent anti-CD20 antibody was previously administered, patients must have received ≥2 doses 11. Documented failure to achieve at least partial response or documented disease progression after response to the most recent treatment regimen 12. Active disease per iwCLL 2018 criteria [1] that requires treatment. At least 1 of the following criteria should be met:     1. Evidence of progressive marrow failure as manifested by the development or worsening of anemia (hemoglobin <10 g/dL) and/or thrombocytopenia (platelets <100,000/μL)     2. Massive (ie, ≥6 cm below the left costal margin), progressive, or symptomatic splenomegaly     3. Massive (ie, ≥10 cm in the longest diameter), progressive, or symptomatic lymphadenopathy     4. Progressive lymphocytosis with an increase of >50% over a 2-month period or a lymphocyte doubling time (LDT) of <6 months. LDT may be obtained by linear-regression extrapolation of absolute lymphocyte count obtained at intervals of 2 weeks over an observation period of 2 to 3 months. In patients with initial blood lymphocyte counts of <30 × 10^9^/L (30,000/μL), LDT should not be used as a single parameter to define indication for treatment. In addition, factors contributing to lymphocytosis or lymphadenopathy other than CLL (eg, infections) should be excluded     5. Autoimmune anemia and/or thrombocytopenia that is poorly responsive to standard therapy     6. B symptoms documented in the patient’s chart with supportive objective measures, as appropriate, defined as ≥1 of the following disease-related symptoms or signs:        1. Unintentional weight loss ≥10% within the previous 6 months before screening        2. Significant fatigue (ECOG performance status ≥2; inability to work or perform usual activities)        3. Fever >100.5°F or 38.0°C for ≥2 weeks before screening without evidence of infection        4. Night sweats for ≥1 month before screening without evidence of infection 13. Negative pregnancy test (urine or serum) for female patients of childbearing potential prior to enrollment 14. Female patients of childbearing potential who are sexually active with a nonsterilized male partner must use highly effective contraception from screening through 2 days after the last dose of acalabrutinib |
| **Exclusion criteria (medical conditions)** |
| 1. Prior malignancy, except for adequately treated basal cell or squamous cell skin cancer, in situ cervical cancer, or other cancer from which the patient had been disease free for ≥2 years or which would not have limited survival to <2 years. Note: These cases must have been discussed with the study physician 2. Life-threatening illness, medical condition, or organ system dysfunction which, in the investigator’s opinion, could have compromised the patient’s safety, interfered with the absorption or metabolism of acalabrutinib, or affected the study outcomes 3. Significant cardiovascular disease such as uncontrolled or symptomatic arrhythmias, congestive heart failure, or myocardial infarction within 6 months of screening, or any class 3 or 4 cardiac disease as defined by the New York Heart Association functional classification 4. Significant screening electrocardiogram (ECG) abnormalities including left bundle branch block, 2^nd^ degree atrioventricular (AV) block type II, 3^rd^ degree AV block, grade ≥2 bradycardia, or average QT interval corrected for heart rate (QTc) from the 3 screening ECGs >480 msec (calculated using Fridericia’s formula) 5. Malabsorption syndrome, disease significantly affecting gastrointestinal (GI) function, or resection of the stomach or small bowel, gastric bypass, symptomatic inflammatory bowel disease, or partial or complete bowel obstruction 6. Known central nervous system involvement of lymphoma/leukemia or leptomeningeal disease 7. Known history of HIV, serologic status reflecting active hepatitis B or C infection, or any uncontrolled active systemic infection    1. Patients who are hepatitis B core antibody positive and surface antigen (HBsAg) negative will need to have a negative hepatitis B virus DNA polymerase chain reaction (PCR) result before enrollment; those who are HBsAg positive or hepatitis B PCR positive will be excluded    2. Patients who are hepatitis C antibody positive will need to have a negative hepatitis C virus RNA PCR result before enrollment; those who are hepatitis C PCR positive will be excluded 8. Major surgery within 4 weeks before first dose of study drug. Note: If a patient had major surgery, they must have recovered adequately from any toxicity and/or complications from the intervention before the first dose of study drug 9. Ongoing drug-induced pneumonitis 10. Uncontrolled autoimmune hemolytic anemia or idiopathic thrombocytopenic purpura 11. Known history of a bleeding diathesis (eg, hemophilia, von Willebrand disease) 12. History of stroke or intracranial hemorrhage within 6 months before the first dose of study drug 13. Known prolymphocytic leukemia or history of (or currently suspected) Richter syndrome (for CLL/small lymphocytic lymphoma) 14. Presence of a GI tract ulcer diagnosed by endoscopy within 3 months prior to screening 15. Uncontrolled active systemic fungal, bacterial, viral, or other infection (defined as exhibiting ongoing signs/symptoms related to the infection and without improvement, despite appropriate antibiotics or other treatment) or ongoing intravenous anti-infective treatment 16. Common Terminology Criteria for Adverse Events grade ≥2 toxicity (other than alopecia, neutropenia, and thrombocytopenia outlined in inclusion criterion #6) continuing from prior anticancer therapy including radiation |

**Online Resource 2.** Dose modification protocol

The actions described in **Online Table 1** should be taken for the following toxicities (according to Common Terminology Criteria for Adverse Events [CTCAE] criteria version 5.0 or higher):

- Grade 4 neutropenia (absolute neutrophil count [ANC] <500/μL) for >7 days (myeloid growth factors are permitted per Consensus of Chinese Experts (version 2015) and use must be recorded on the electronic case report form [eCRF]).
- Grade 3 platelet decreases in the presence of clinically significant bleeding
- Grade 4 platelet decreases
- Grade 3 or 4 nausea, vomiting, or diarrhea, if persistent despite optimal antiemetic and/or anti-diarrheal therapy
- Any other grade 4 toxicity or unmanageable grade 3 toxicity

If the toxicity resolves or reverts to ≤CTCAE grade 1 or baseline within 28 days of onset and the subject is showing clinical benefit, treatment with acalabrutinib may be restarted using the rules described below for dose modifications (see **Online Table 1**). Whenever possible, any dose adjustment of acalabrutinib should be discussed between the investigator and the sponsor before implementation. The appropriate clinic staff should dispense the study drug for the new dose level and instruct the patient/caregiver about the change in dose level. Any changes to the dosing regimen must be recorded in the appropriate eCRF.

If a dose reduction is necessary, the study intervention will be administered as described in **Online Table 1**. Appropriate and optimal treatment of the toxicity is assumed prior to considering dose modifications. Prior to discontinuation of study intervention due to toxicities, please consult with the study physician.

**Online Table 1.** Dose modifications for toxicity

| **Occurrence** | **Action** |
| --- | --- |
| 1^st^ – 2^nd^ | Hold acalabrutinib until recovery to grade ≤1 or baseline; may restart at original daily dose level (100 mg BID) |
| 3^rd^ | For non-hematologic adverse events (AEs), discontinue acalabrutinib. For thrombocytopenia with significant bleeding, discontinue acalabrutinib. For other hematologic AEs, upon recovery to grade ≤1 or baseline, restart at 100 mg QD |
| 4^th^ | For hematologic AEs, discontinue acalabrutinib |

**Online Resource 3.** Progression-free survival (A) and duration of response (B) as assessed by investigators. ^a^Two deaths were reported during the study: 1) cardiac arrest in a 73-year-old patient on study day 34 (2 days after the last dose of study drug) and possibly related to study drug, and 2) hemorrhagic shock in a 55-year-old patient on study day 66 (50 days after discontinuing study drug) and unclear about the relationship to study treatment. DOR, duration of response; PFS, progression-free survival.


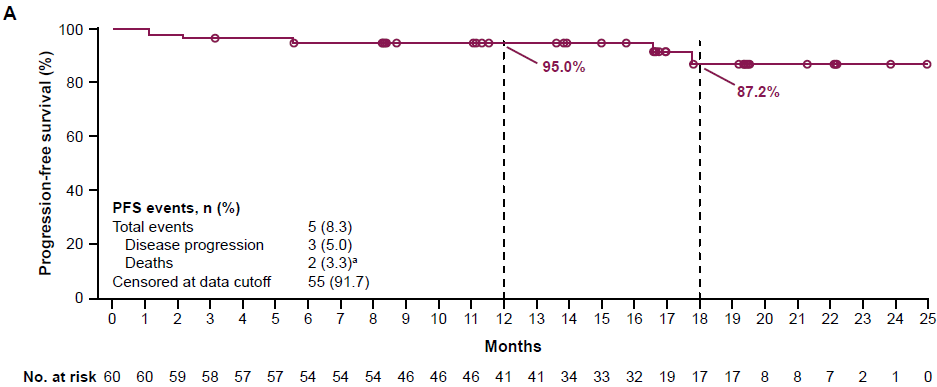


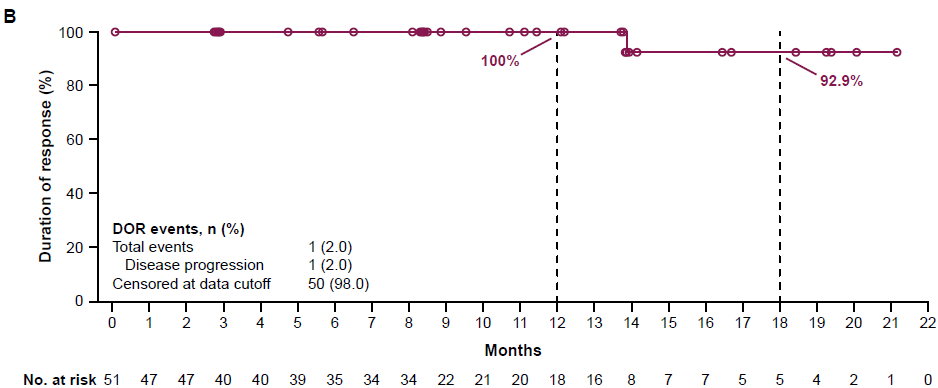


**Online Resource 4.** Subgroup analysis of PFS rate at 12 months by BICR. BICR, blinded independent central review; CI, confidence interval; ECOG, Eastern Cooperative Oncology Group; IGHV, immunoglobulin heavy chain variable region genes; PFS, progression-free survival.

^a^Age when informed consent was signed; ^b^At baseline.


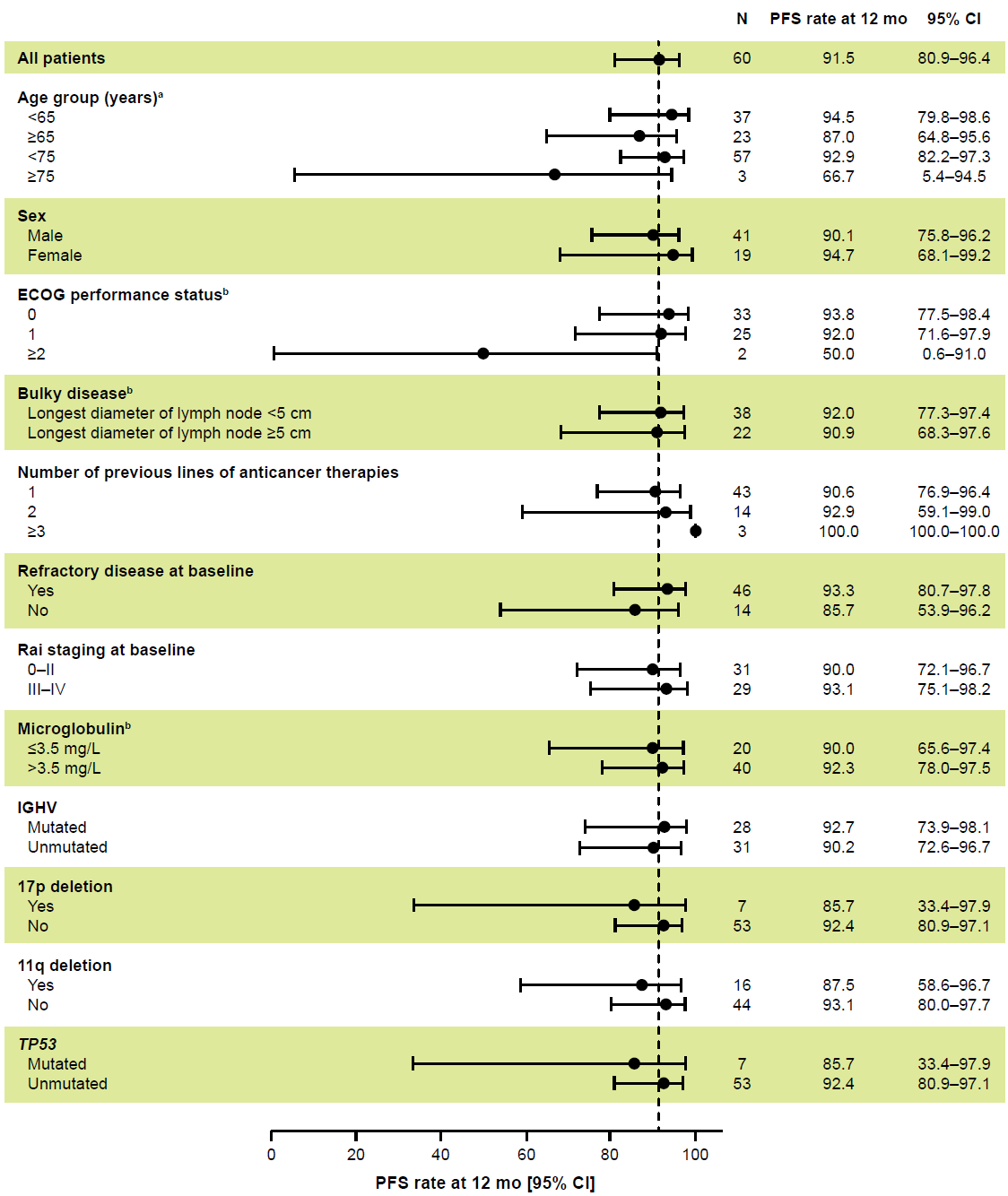


**Online Resource 5.** Serious adverse events

|  | No. (%) of patients  (N=60)^a^ |
| --- | --- |
| Any SAE | 9 (15.0) |
| Pneumonia | 2 (3.3) |
| Anemia | 1 (1.7) |
| Anal abscess | 1 (1.7) |
| Arteriosclerosis coronary artery | 1 (1.7) |
| Asthma | 1 (1.7) |
| Cardiac arrest | 1 (1.7) |
| Coronary artery disease | 1 (1.7) |
| Febrile neutropenia | 1 (1.7) |
| Paraneoplastic pemphigus | 1 (1.7) |
| Pelvic mass | 1 (1.7) |
| Rectal neoplasm | 1 (1.7) |

SAE, serious adverse event

^a^Patients with multiple events in the same preferred term are counted only once in that preferred term. Patients with events in more than one preferred term are counted once in each of those preferred terms.

**Online Resource 6.** Efficacy and safety comparison of four BTK inhibitors approved in China for patients with R/R CLL/SLL

|  | **Acalabrutinib** | **Ibrutinib [2]** | **Zanubrutinib [3]** | **Orelabrutinib [4]** |
| --- | --- | --- | --- | --- |
| Trial identifier | NCT03932331 | NCT01973387 | NCT03206918 | NCT03493217 |
| Number of patients | 60 | 106 (86% Chinese) | 91 | 80 |
| Phase | 1/2 | 3 | 2 | 2 |
| Median number of prior lines of therapy | 1 | NA  (mean=2.0) | 1 | 1 |
| Median follow-up | 20.2 mo | 17.8 mo | 15.1 mo | 32.3 mo |
| ORR (CR+PR+PRL) | 87% | 68% | 85% | 93% |
| PFS/EFS | 12-mo PFS: 92%  18-mo PFS: 79% | 18-mo PFS: 74% | 12-mo EFS: 87% | 30-mo PFS: 71% |
| OS | 18-mo OS: 97% | 24-mo OS: 80% | 12-mo OS: 96% | 24-mo and 30-mo OS: 81% |
| Select any-grade AEs (grade ≥3) |  |  |  |  |
| Infections | 60.0% (11.7%) | 68.3% (NA) | 86.9% (37.4%) | NA |
| Diarrhea | 15.0% (1.7%) | 33.7% (3.8%) | 19.8% (2.2%) | 10.0% (1.3%) |
| Atrial fibrillation | 0 | 5.8% (NA) | 0 | 0 |
| Hypertension | 5.0% (0) | 5.8% (1.9%) | 9.9% (2.2%) | 5.0% (1.3%) |
| Hemorrhage | 40.0% (0) | 28.8% (NA)^a^ | 62.7% (2.2%) | 20.0% (NA)^b^ |

AE, adverse event; CLL, chronic lymphocytic leukemia; CR, complete response; EFS, event-free survival; ORR, overall response rate; OS, overall survival; PFS, progression-free survival; PR, partial response; PRL, partial response with lymphocytosis; NA, not available; R/R, relapsed/refractory; SLL, small lymphocytic lymphoma.

^a^Major hemorrhage was reported in 2.9% of patients receiving ibrutinib.

^b^Two (2.5%) patients had major hemorrhage events (serious, any-grade central nervous system bleeding, or grade ≥3 events).

**PLAIN LANGUAGE SUMMARY**

Chronic lymphocytic leukemia (CLL) is a rare type of cancer that develops from white blood cells. Acalabrutinib is a treatment approved for patients with CLL. Most of the studies used to support the approval of acalabrutinib by governmental agencies were done mostly with Caucasian patients. This is the first study to evaluate acalabrutinib exclusively in Chinese patients with previously treated CLL. Patients were examined and interviewed to collect data about side effects (safety evaluation) and had imaging studies at predetermined periods to assess how the tumors were responding to the treatment (efficacy evaluation). A large majority of patients with CLL had good responses to acalabrutinib that were long-lasting. The treatment was considered safe, with tolerable side effects. The results in this Chinese population of patients were similar to the key global study in a primarily Caucasian population of patients with previously treated CLL. Patients from different races or ethnic backgrounds can react differently to certain medications, so it is useful to collect safety and efficacy data for various populations. This study demonstrated that the efficacy and safety of acalabrutinib were comparable between Chinese and Caucasian patients with previously treated CLL.

**References**

1. Hallek M, Cheson BD, Catovsky D, Caligaris-Cappio F, Dighiero G, Döhner H, Hillmen P, Keating M, Montserrat E, Chiorazzi N, Stilgenbauer S, Rai KR, Byrd JC, Eichhorst B, O'Brien S, Robak T, Seymour JF, Kipps TJ (2018) iwCLL guidelines for diagnosis, indications for treatment, response assessment, and supportive management of CLL. Blood 131 (25):2745-2760. doi:10.1182/blood-2017-09-806398

2. Huang X, Qiu L, Jin J, Zhou D, Chen X, Hou M, Hu J, Hu Y, Ke X, Li J, Liang Y, Liu T, Lv Y, Ren H, Sun A, Wang J, Zhao C, Salman M, Sun S, Howes A, Wang J, Wu P, Li J (2018) Ibrutinib versus rituximab in relapsed or refractory chronic lymphocytic leukemia or small lymphocytic lymphoma: a randomized, open-label phase 3 study. Cancer Med 7 (4):1043-1055. doi:10.1002/cam4.1337

3. Xu W, Yang S, Zhou K, Pan L, Li Z, Zhou J, Gao S, Zhou D, Hu J, Feng R, Huang H, Ji M, Guo H, Huang J, Novotny W, Feng S, Li J (2020) Treatment of relapsed/refractory chronic lymphocytic leukemia/small lymphocytic lymphoma with the BTK inhibitor zanubrutinib: phase 2, single-arm, multicenter study. J Hematol Oncol 13 (1):48. doi:10.1186/s13045-020-00884-4

4. Xu W, Zhou K, Wang T, Yang S, Liu L, Hu Y, Zhang W, Ding K, Zhou J, Gao S, Xu B, Zhu Z, Liu T, Zhang H, Hu J, Ji C, Wang S, Xia Z, Wang X, Li Y, Song Y, Ma S, Tang X, Zhang B, Li J (2023) Orelabrutinib in relapsed or refractory chronic lymphocytic leukemia/small lymphocytic lymphoma patients: multi-center, single-arm, open-label, phase 2 study. Am J Hematol 98 (4):571-579. doi:10.1002/ajh.26826
